# Tilapia (*Oreochromis niloticus*) as a Putative Reservoir Host for Survival and Transmission of *Vibrio cholerae* O1 Biotype El Tor in the Aquatic Environment

**DOI:** 10.3389/fmicb.2019.01215

**Published:** 2019-05-31

**Authors:** Yaovi Mahuton Gildas Hounmanou, Robinson H. Mdegela, Tamegnon Victorien Dougnon, Henry Madsen, Jeffrey H. Withey, John E. Olsen, Anders Dalsgaard

**Affiliations:** ^1^Department of Veterinary and Animal Sciences, Faculty of Health and Medical Sciences, University of Copenhagen, Copenhagen, Denmark; ^2^Department of Veterinary Medicine and Public Health, College of Veterinary Medicine and Biomedical Sciences, Sokoine University of Agriculture, Morogoro, Tanzania; ^3^Research Unit in Applied Microbiology and Pharmacology of Natural Substances, Research Laboratory in Applied Biology, Polytechnic School of Abomey-Calavi, University of Abomey-Calavi, Abomey-Calavi, Benin; ^4^Department of Biochemistry, Microbiology, and Immunology, Wayne State University School of Medicine, Detroit, MI, United States

**Keywords:** *Vibrio cholerae*, tilapia, cholera transmission, microbial ecology, reservoirs

## Abstract

Studies have reported the occurrence of *Vibrio cholerae* in fish but little is known about the interaction between fish and toxigenic *V. cholerae* as opposed to phytoplankton, which are well-established aquatic reservoirs for *V. cholerae*. The present study determined the role of tilapia (*Oreochromis niloticus*) as a reservoir host for survival and transmission of *V. cholerae* in aquatic environments. Three experiments were performed with one repetition each, where *O. niloticus* (∼2 g) kept in beakers were inoculated with four *V. cholerae* strains (5 × 10^7^ cfu/mL). Firstly, infected tilapia were kept in stagnant water and fed live brine shrimp (*Artemia salina*) larvae daily. Secondly, infected tilapia were kept without feeding and water was changed every 24 h. Thirdly, infected tilapia were fed and water was renewed daily. Infected tilapia and non-infected controls were sacrificed on days 1, 2, 3, 7, and 14 post-inoculation and *V. cholerae* were enumerated in intestinal content and water. Another experiment assessed the transmission of *V. cholerae* from infected to non-infected tilapia. The study revealed that El Tor biotype *V. cholerae* O1 and *V. cholerae* non-O1 colonized tilapia intestines and persisted at stable concentrations during the second week of the experiment whereas the Classical biotype was undetectable after 1 week. In stagnant water with feeding, *V. cholerae* counts dropped to 10^5^ cfu/ml in water and from 10^7^ to 10^4^ cfu/intestine in fish after 14 days. When water was renewed, counts in water decreased from 10^7^ to 10^3^ cfu/ml and intestinal counts went from 10^6^ to 10^2^ cfu/intestine regardless of feeding. All strains were transmitted from infected to naïve fish after 24 h of cohabitation. Tilapia like other fish may play an essential role in the survival and dissemination of *V. cholerae* O1 in aquatic environments, e.g., the seventh pandemic strains mostly. In this study, tilapia were exposed to high concentrations of *V. cholerae* to ensure initial uptake and follow-up studies with lower doses resembling natural concentrations of *V. cholerae* in the aquatic environment are needed to confirm our findings.

## Introduction

*Vibrio cholerae* is one of the longest recognized human infectious pathogens, yet there is still much to clarify on the emergence and transmission of cholera, the disease for which *V. cholerae* is the causative agent. *V. cholerae* O1 and O139 are the only serogroups causing cholera, with the leading strains being the toxigenic *V. cholerae* O1 El Tor and Classical biotypes (Dalsgaard et al., 2001). The Classical biotype, however, has not been implicated in cholera outbreaks for several decades and has become extremely rare, if not extinct, in the aquatic environment since the beginning of the seventh cholera pandemic (Safa et al., 2006; Nag et al., 2018). The main virulence factor in humans expressed by all biotypes is cholera toxin. The intestinal colonization of *V. cholerae* in humans requires production of the cholera toxin co-regulated pilus (TCP), whose main transcription activator is ToxT (Faruque et al., 1998; Sanchez and Holmgren, 2011). *V. cholerae* O1 biotype El Tor that lacks active *tox*T can therefore be regarded as non-toxigenic. *V. cholerae* non-O1/O139 strains are ubiquitous in aquatic environments and rarely produce cholera toxin, but can cause sporadic diarrhea (Hounmanou et al., 2016). Most *V. cholerae* non-O1 do not contain *tcp*A (regulated by ToxT), but if present, and the role of *tox*T remains the same which is to regulate the transcription of TCP. In this study, a *tox*T mutant of *V. cholerae* O1 El Tor served to assess whether the lack of transcription of *tcp*A (regulated by ToxT) would affect colonization in tilapia.

Fish are potential carriers for *V. cholerae* and the occurrence of toxigenic and non-toxigenic strains of *V. cholerae* in tilapia (*Oreochromis niloticus*) and African catfish (*Clarias gariepinus*) has been reported during non-cholera outbreak periods (Hounmanou et al., 2016). *V. cholerae* non-O1 has also been isolated in carps (*Rastrineobola argentea*) from Lake Tanganyika (Nyambuli et al., 2018). A study in Bangladesh indicated that fish could serve as a potential vehicle for *V. cholerae* transmission to humans (Hossain et al., 2018), and genetic analysis of *V. cholerae* from a cholera outbreak in Zanzibar suggested that marine fish were implicated in pathogen transmission (Rabia et al., 2017).

In sub-Saharan Africa, where the seventh cholera pandemic has a high impact in terms of morbidities and mortalities (Mengel et al., 2014; Weill et al., 2017), frequent epidemics occur around the African Great Lakes Region (AGLR), where fishing, and fish processing represent an essential socio-economic activity (Nkoko et al., 2011; Plisnier et al., 2015; Ajayi and Smith, 2018). Association between cholera and the aquatic environment is well-established in the AGLR (Urassa et al., 2009; Reyburn et al., 2011). Well-recognized aquatic reservoirs of *V. cholerae* include phytoplanktons, zooplanktons, algae, and cyanobacteria. The role of fish as a reservoir host, however, remain speculative because the mere presence of *V. cholerae* in fish is not sufficient to confirm that fish is a true reservoir host providing multiplication and persistence of the pathogen (Tamplin et al., 1990; Halpern et al., 2004; Islam et al., 2015; Halpern and Izhaki, 2017).

Studies conducted using zebrafish as models indicated that they can be colonized by *V. cholerae* and can transmit the bacteria to naïve zebrafish via excretion (Runft et al., 2014; Mitchell et al., 2017). This therefore calls for further studies to explore the fate of *V. cholerae* in wild caught fish like tilapia.

In the present experimental study, we worked with one of the common edible fish species around the AGLR, namely tilapia (*O*. *niloticus*). The aim was to determine the role of tilapia in the survival of *V. cholerae* in the aquatic environment and in transmitting the pathogen. The results suggest that *V. cholerae* O1 El Tor (causing the seventh cholera pandemic) do survive in tilapia, which may have important implications in the epidemiology of the ongoing cholera epidemic around the AGLR.

## Materials and Methods

### Bacterial Strains Used in Tilapia Experiments

*Vibrio cholerae* O1 El Tor (strain E7946) and Classical (strain O395) biotypes used in this study were clinical strains obtained from a laboratory collection (Table 1). They are all streptomycin resistant, which allowed for the use of selective isolation procedures during the experiment. An environmental *V. cholerae* non-O1 was isolated from carps (*Rastrineobolla agentea*) in Lake Victoria, Tanzania, and confirmed streptomycin resistant by disc diffusion following standard methods of isolation and identification of *V. cholerae* (Hounmanou et al., 2016). The actual serotype of this non-O1 strain was not determined. The strain was included in the study to assess whether colonization in tilapia differs between clinical strains of serogroup O1 and the environmental *V. cholerae* non-O1 strains. *V. cholerae* O1 strain JW612, a *tox*T mutant was included to test whether the lack of transcription of *tcp*A (regulated by *tox*T) would affect colonization of the tilapia (Table 1).

**Table 1:** Streptomycin-resistant *V. cholerae* strains used in this study.

Strain ID	Strain characteristics	Source
O395	*V. cholerae* O1, classical biotype	Runft et al., 2014
E7946	*V. cholerae* O1, El Tor biotype	Runft et al., 2014
JW612	*V. cholerae* O1 Δ*toxT* (mutant of E7946)	Runft et al., 2014
*V. cholerae* non-O1	*Ctx*-negative, *V. cholerae* non-O1 strain isolated from carps in Lake Victoria	This study


### Elimination of Natural *V. cholerae* in Tilapia Prior to Experiment

We used tilapia (*O*. *niloticus*) juveniles (approximately 2 g) obtained from a hatchery at the Sokoine University of Agriculture in Morogoro, Tanzania where the experiments were performed. About 20 tilapia juveniles were placed in beakers (2,000 mL) containing autoclaved tap water with constant aeration. To ensure that the tilapia juveniles did not contain environmental *V. cholerae*, intestinal samples of five tilapia per beaker were plated on thiosulfate-citrate-bile salts-sucrose agar (TCBS) (Oxoid Limited, Hampshire, United Kingdom) after 24, 48, and 72 h. This was followed by a species-specific PCR for the *omp*W gene (Nandi et al., 2000) using DNA from isolates recovered on TCBS agar and from samples enriched in APW. Water samples from the beakers were also cultured for *V. cholerae*. No *V. cholerae* colonies were detected in any water and fish gut samples and none of the samples produced the 588 bp band expected for the *omp*W gene. Tilapia juveniles confirmed negative for *V. cholerae* were used in the experiment.

### Exposure of Tilapia to *V. cholerae* Inoculated in Water

The four *V. cholerae* strains used in the experiments (Table 1) were first grown in Luria-Bertani (LB) broth (Difco, Becton Dickinson and company, Maryland, United States) at 37°C for 24 h with agitation. The growth curves of the strains revealed that the overnight cultures (24 h at 37°C) of all the strains reached on average 10^10^ cfu/mL (data not shown). Following procedures described for zebrafish models (Runft et al., 2014; Mitchell et al., 2017), the *V. cholerae* strains were grown overnight in tubes containing 10 ml LB. Tubes with the overnight bacterial cultures were centrifuged at 5,000 *g* for 2–3 min and the pellets with bacteria were washed twice in normal saline solution (sterile water + 0.9% NaCl). Bacterial cells collected from 10 LB tubes were suspended in one mL normal saline/tube and added to beakers (aquarium) containing 2,000 mL of autoclaved tap water and 20 tilapia juveniles. Thus, the count of each *V. cholerae* strain was about 5 × 10^7^ cfu/mL in each beaker, similar to concentrations used in the zebrafish models (Runft et al., 2014). This concentration is high compared to the expected concentration of *V. cholerae* in the natural aquatic environment. However, because gavage is not a natural route of administration in fish, we exposed tilapia to the test strains of *V. cholerae* by immersion in the beakers; a high dose of inoculum was therefore needed to ensure an uptake of the test strains. The beakers were kept at room temperature (25°C) for 2 weeks with constant aeration by an air pump. The experiment included four beakers with the *V. cholerae* strains (Table 1) and another beaker containing tilapia juveniles were given 1 mL sterile normal saline solution with no *V. cholerae* (control). Three tilapia juveniles were sacrificed 1, 2, 3, 7, and 14 days after inoculation of the *V. cholerae* strains. Fish were infected similarly, in all experiments.

### Experimental Design

We conducted three different experiments with two replicates of each. Each experiment was repeated 2 weeks after the end of the first experiment to have a biological replicate that ensures the validity of the study. In each experiment, tilapia were inoculated with 5 × 10^7^ cfu/mL of various *V. cholerae* strains (Table 1) by immersion in beakers as previously described (Mitchell et al., 2017). Tilapia (∼2 g) were sacrificed on days 1, 2, 3, 7, and 14 post-exposure and the concentration of *V. cholerae* in water and intestinal content was enumerated as was the water turbidity based on OD_600_ water measurements. The beakers with tilapia, but no *V. cholerae* served as controls and one mL of sterile normal saline solution was added at the onset of the experiment.

In Experiment 1, tilapia were starved for 24 h before the experiment. Two hours after the inoculation of the *V. cholerae* strains, feeding was initiated with fresh hatched brine shrimp (*Artemia salina*) (JBL GmbH & Co., Neuhofen, Germany) served twice a day until termination of the experiment. The brine shrimp were hatched from dry eggs in sterile water and did not contain *V. cholerae* as shown by lack of yellow bacterial colonies when grown on TCBS agar plates. Water in the beakers remained unchanged until the experiment was terminated. This experiment aimed to determine the colonization, the survival and shedding of the *V. cholerae* test strains in tilapia in stagnant water. The control for this experiment was a beaker with tilapia fed with the same brine shrimp and inoculated with sterile normal saline. We chose live feed because of the size of the juveniles being used in the study and also to avoid commercial feeds which may increase water fouling in the beakers.

In Experiment 2, water in the beakers was replaced daily (every 24 h) with fresh sterile water of the same volume (2,000 mL) and fish were not fed. When the water was changed, fish were removed from the initial beaker then washed twice in sterile tap water to remove external *V. cholerae* by rubbing their surface before placing them in a new beaker with sterile water. Infection procedures were the same as in Experiment 1 but this experiment aimed to assess the impact of feeding and water renewal on the survival and excretion of *V. cholerae* in tilapia in the absence of feeding.

In Experiment 3, water was changed like in Experiment 2, but fish were fed brine shrimp free of *V. cholerae* like in the Experiment 1. Tilapia was exposed to *V. cholerae* by immersion as in the other experiments. This experiment aimed to differentiate between the impact of feeding and water exchange on the survival and excretion of *V. cholerae* in tilapia.

### Enumeration of *V. cholerae*

Fish were collected every morning and sacrificed. In Experiments 2 and 3 (where water was renewed), fish, and water samples were taken from the beakers before water renewal. After the tilapia juveniles (three fish were collected per time point) were sacrificed, the intestinal content was aseptically removed using sterile scissors and metal blades, and the content placed into 1 mL of sterile normal saline. After homogenization of the intestinal contents by crushing and shaking, serial decimal dilutions of the homogenate were made and 10 μL of dilutions were subsequently spread onto Luria-Bertani Agar (LA) (Difco) plates containing streptomycin (100 μg/mL). One mL water samples from the beakers were collected at each time point and diluted and plated on LA as described for the intestinal content samples. When we obtained 30 or less colonies on LA plates containing streptomycin, all isolates were re-streaked on TCBS agar for confirmation. With higher colony numbers, the identity of at least 30 colonies appearing on the LA plates was confirmed on TCBS agar. Selected El Tor and Classical isolates were collected from TCBS agar plates and confirmed as *ctxA*-positive by PCR to verify that they did not lose their virulence. In Experiments 2 and 3, colony counts were lower due to the daily change of water and, therefore, 100 μL of each dilution was plated on LA. Colony forming units per fish intestine or mL of water were calculated using counts from all plates based on dilutions with valid counts divided by the sum of offset values. Thus in Experiment 1, the detection limit for one sample (fish intestine or water sample) was 900 cfu (2.95 on a logarithmic scale with base 10) per intestine or per mL of water sample and 150 cfu per intestine for six fish samples combined and 450 cfu pr mL for two water samples. For Experiments 2 and 3 where 100 μL was plated, the detection limit for an individual sample was 9 cfu (1.96 on Log_10_ scale). At each time point intestines from three tilapia were analyzed individually for *V. cholerae*.

The use of high concentrations of *V. cholerae* could increase stress in the fish causing excretion of more waste particles which was evaluated by measuring the optical density values of the water. Like in the zebrafish models (Mitchell et al., 2017), the optical density (OD_600_) of 1 mL water sample was read at 600 nm in a spectrophotometer using normal saline as the blank. Optical density was measured also in the control groups and at different time points during the 14 days of experiment. OD values of the beaker water were measured at each time point along with *V. cholerae* counts in intestine and water samples.

### Assessing Transmission of *V*. *cholerae* Within Tilapia Populations

Ten tilapia juveniles of ∼4 g were exposed to fresh overnight cultures of the *V. cholerae* strains (Table 1) in beakers containing 1 L autoclaved tap water as described above (approximately 5 × 10^7^ cfu/mL). After 6 h of exposure, a time which previously was found sufficient to allow colonization of *V. cholerae* in zebrafish (Runft et al., 2014), the tilapia juveniles were washed in autoclaved tap water twice to remove any external *V. cholerae* present on the fish body. The juveniles were then placed in another beaker with sterile water containing ten naive tilapia juveniles of ∼2 g with the smaller size allowing differentiation from the larger 4 g fish. After 24 h of cohabitation, four of the naïve tilapia juveniles were sacrificed per beaker and intestinal *V. cholerae* populations were enumerated as described above.

### Statistical Analyses

*Vibrio cholerae* counts (x) were calculated as total count per fish intestine (fish samples) or total count per mL (water samples) for the two repeated trials. Comparisons of bacterial counts in fish samples [log_10_(x+1)] between strains and over time was done using multiple linear regression where also the interaction between strain and time was assessed. Repetition was not a significant predictor of bacterial counts neither when tested alone nor in the full model and therefore it was left out of the final analysis. *V. cholerae* counts in water samples were not compared in a similar manner as there was only one sample for each repetition, strain and time point, but those counts were correlated with average counts in the fish using linear regression. Model assumptions were verified using normal probability plot of standardized residuals and histogram of residuals. Homoskedatiscity was checked using rvf-plot plus Breusch-Pagan/Cook-Weisberg test for heteroskedasticity. *P*-values < 0.05 were taken to indicate significant differences in Stata (Version 12, StataCorp, College Station, TX, United States). Bacterial counts from transmission experiments enumerated 24 h post exposure were only strain dependent. Therefore, one-way ANOVA was performed to compare mean counts for the two trials among strains.

## Results

### Survival of *V. cholerae* in Tilapia Kept in Stagnant Water and Given Live Feed

**FIGURE 1:**
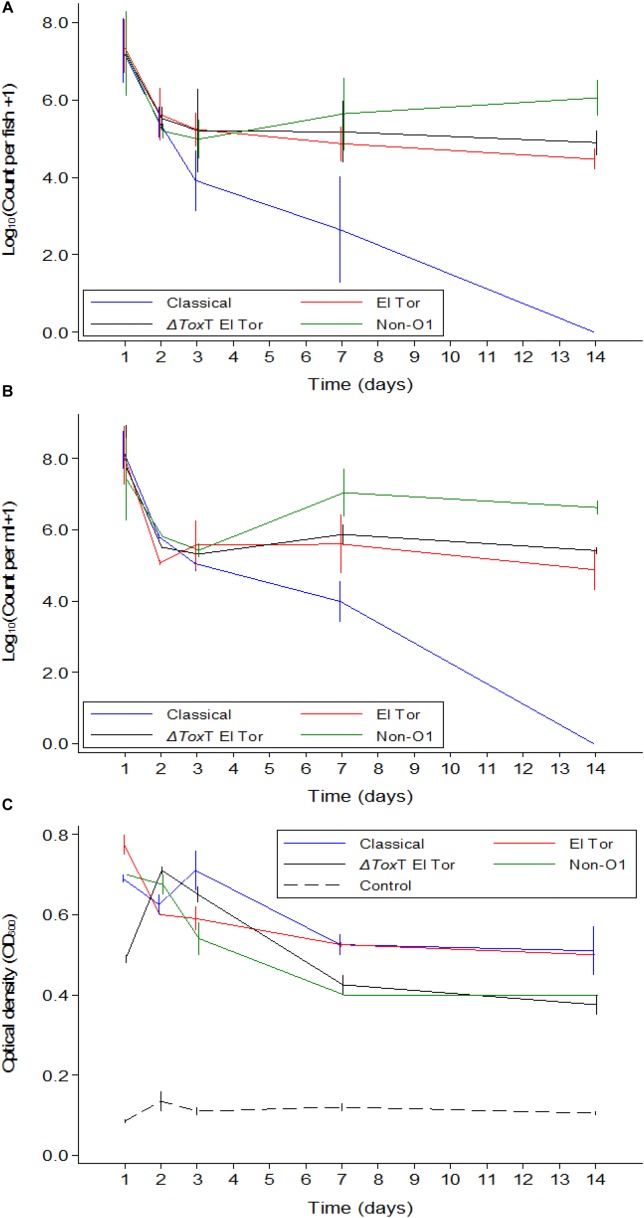
Mean counts of *V. cholerae* from two repetitions in tilapia intestines **(A)** count per fish, in water **(B)** count per mL, and absorbance of water **(C)** over time when tilapia were kept in the same water and given live feed. Error bars indicate 95% CL. Each strain is slightly off its exact *x*-value to allow distinction of the error bars. Relevant statistical differences between strains and time points are indicated in the text.

In Experiment 1, tilapia were exposed to 5 × 10^7^ cfu/mL of *V. cholerae* as described in the Section “Materials Methods.” They fish were kept in stagnant water and fed brine shrimp for the 2 weeks duration of the experiment. Tilapia juveniles exposed to *V. cholerae* were found to be colonized by *V. cholerae* within 24 h after exposure, with average intestinal counts for all test strains varying between 10^7^ cfu/intestine on day 1 post infection to 10^5^ cfu/intestine 14 days after infection. *V. cholerae* counts declined over time for all four strains (Figure 1A), with a significant interaction seen between time and strain (*p* < 0.001). There was no difference in concentration of different test *V. cholerae* strains for the first 2 days. However, 3 and 7 days after exposure, counts were lower for the Classical biotype than those of the *V. cholerae* non-O1 strain (*p* < 0.05 – *p* < 0.001 depending on the day). One fish exposed to the *V. cholerae* O1 Classical biotype did not contain *V. cholerae* on day 7 (i.e., below the detection limit of 2.95 on a log_10_-scale). After 14 days, all fish exposed to the *V. cholerae* O1 Classical biotype no longer contained detectable levels of this strain. Both *V. cholerae* O1 El Tor and a Δ*tox*T *V. cholerae* O1 El Tor, had lower counts than the *V. cholerae* non-O1 strain (*p* < 0.05). Except for the Classical biotype strain, strains survived in tilapia for 2 weeks at constant levels. No *V. cholerae* was detected in the uninfected control tilapia for the duration of the experiment.

**FIGURE 2:**
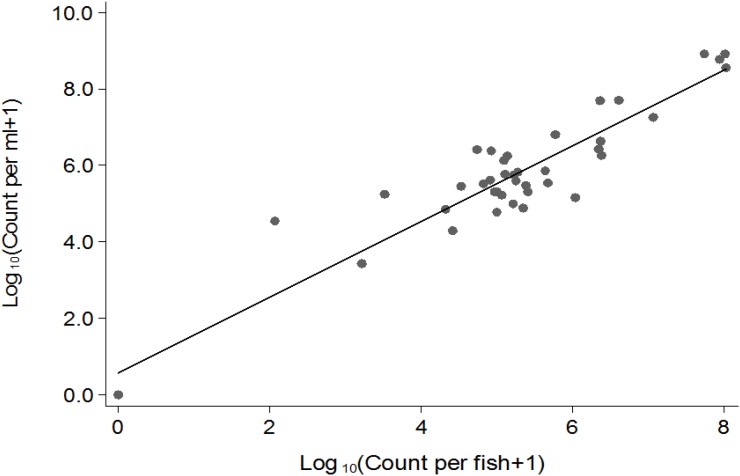
Correlation between Log10 (cfu) in fish (*x*) and in water (*y*).

*Vibrio cholerae* numbers in water were similar to numbers in fish intestinal content. The Classical biotype was not detected in water 1 week after exposure (Figure 1B). Counts in water correlated with average counts in the fish intestine (Figure 2). However, the average *V. cholerae* counts in water varied between 10^8^ cfu/mL on day 1 to 10^6^ cfu/mL 14 days after inoculation of test strains and was higher than those observed in fish intestines. No *V. cholerae* was isolated from water in the control beakers.

Optical density of water from beakers with fish exposed to *V. cholerae* strains was considerably higher than the density in aquaria with unexposed fish, even for the Classical biotype strain after it was no longer detectable. Significant predictors of optical density when analyzing only the four *V. cholerae* strains were strain (*p* < 0.05) and time (*p* < 0.001). The Classical and El Tor biotypes did not differ when adjusting for time, while the Δ*toxT* mutant of El Tor, and the non-O1 strains both differed from the Classical strain but did not differ between themselves. Optical water densities were lower at day 7 and 14 than during day 1 (Figure 1C).

### Survival of *V. cholerae* in Tilapia When Water Was Changed Daily and Tilapia Were Not Fed

**FIGURE 3:**
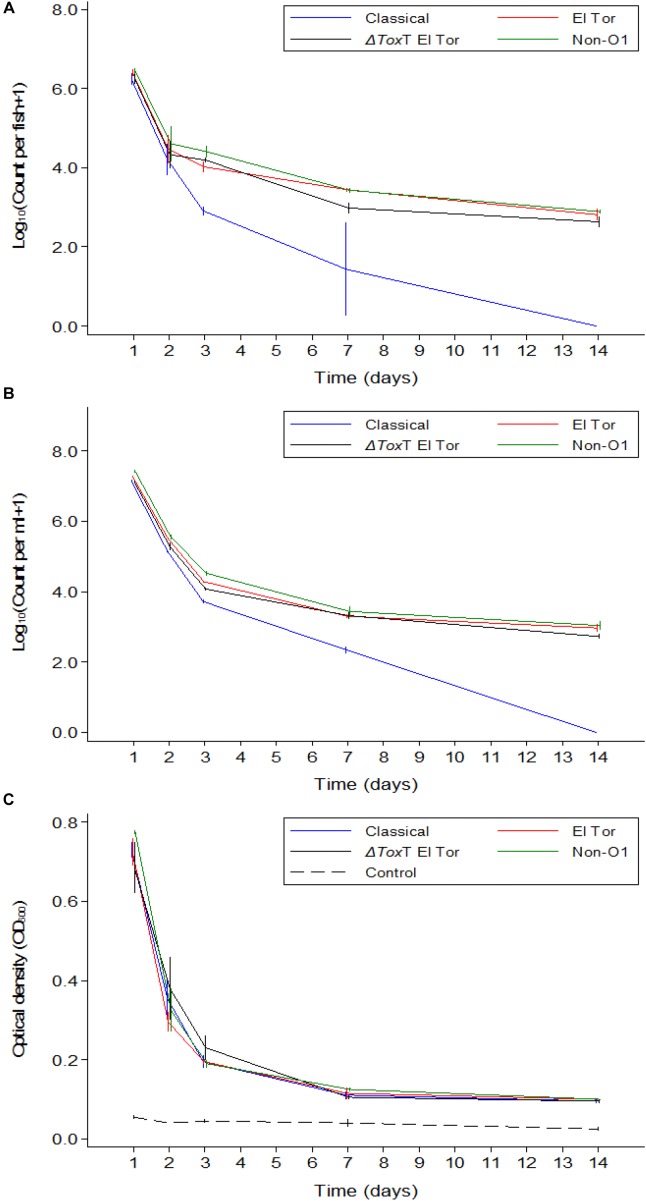
Mean counts of *V. cholerae* from two repetitions in tilapia intestines **(A)** count per fish, in water **(B)** count per mL, and absorbance of water **(C)** over time when aquarium water was changed daily and tilapia were not fed. Error bars indicate 95% CL. Each strain is slightly off its exact *x*-value to allow distinction of the error bars. Relevant statistical differences between strains and time points are indicated in the text.

In Experiment 2, fish were exposed to *V. cholerae* as described above. However, in contrast to Experiment 1, the tilapia were not fed and the water was changed daily. Thus, after the initial infection only *V. cholerae* that multiplied in the intestine and excreted by the fish would be detected in the water. With daily water exchange and absence of feeding, tilapia were still colonized by all strains of *V. cholerae* 24 h post infection with average counts around 10^6^ cfu/intestine (Figure 3A). Up to 2 days post infection, there was no difference in colonization levels between strains (*p* > 0.05). However, from day 3 post infection, the concentration of the Classical biotype strain decreased significantly in the fish intestines and was undetectable after 1 week (*p* < 0.001). Despite the absence of feeding and with the constant daily water exchange, tilapia remained colonized with the three other strains of *V. cholerae* until the end of the 1 weeks, but the counts dropped significantly from day to day, most significantly during the first 7 days (*p* < 0.05). No *V. cholerae* growth was detected in the uninfected control group. During the second week, 5–15% mortality was recorded in all beakers probably due to starvation.

In Experiment 2, *V. cholerae* concentrations in water were similar to those in the intestine, varying from 10^7^ cfu/mL on day one post infection to 10^3^ cfu/mL 14 days after inoculation, with significant daily decreases (*p* < 0.05). Like in the fish intestine, the Classical biotype strain could no longer be detected in the water after 1 week (Figure 3B). The other three strains remained present in the water despite the daily water replacement with fresh sterile water, suggesting continuous *V. cholerae* multiplication, and excretion by the fish. No *V. cholerae* was detected in the uninfected fish from the control beakers.

Overall, when tilapia were starved and water was exchanged daily, the optical density of water in beakers containing infected tilapia remained significantly higher than in the control beakers where fish were not infected (*p* < 0.05). The difference was more pronounced in the first week (*p* < 0.001); however, in the second week of the experiment, excretion levels decreased as the OD values from infected groups became statistically similar to the OD values of the uninfected control even though the numbers were higher than that of the control (*p* > 0.05). There was no significant difference between the four strains in terms of excretion at any time point (*p* > 0.05). Despite the absence of the Classical biotype strain in the second week, the OD values in that aquarium remained similar to that of the other strains (Figure 3C).

### Survival of *V. cholerae* in Tilapia When Water Was Changed Daily and Tilapia Were Given Live Feed

**FIGURE 4:**
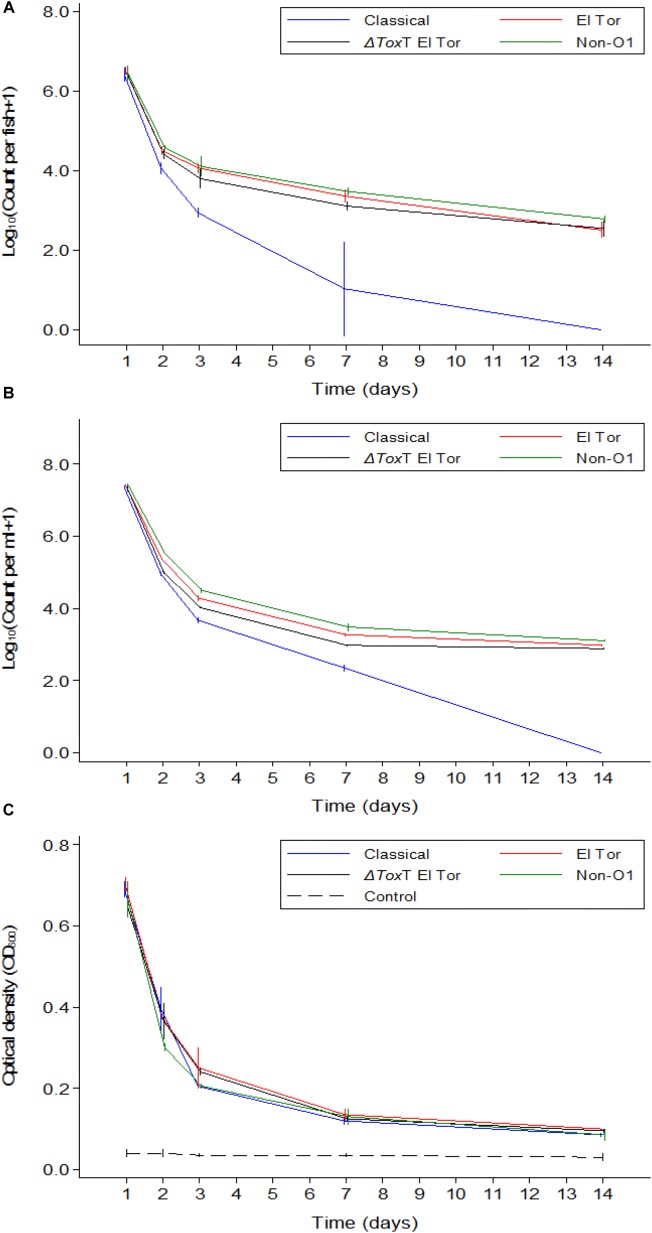
Mean counts of *V. cholerae* from two repetitions in tilapia intestines **(A)** count per fish, in water **(B)** count per mL, and absorbance of water **(C)** over time when aquarium water was changed daily and tilapia are given live feed. Error bars indicate 95% CL. Each strain is slightly off its exact *x*-value to allow distinction of the error bars. Relevant statistical differences between strains and time points are indicated in the text.

In Experiment 3, fish were again infected with *V. cholerae* and water was renewed daily. In contrast to Experiment 2, these fish were also fed daily. *V. cholera*e counts in tilapia intestines, counts in water, and the water OD measurements showed similar values and trends as those recorded in Experiment 2 where tilapia were starved (Figure 4A–C). However, there was no fish mortality in Experiment 3 as compared to the Experiment 2 where fish were not fed.

### Comparison of *V. cholerae* Counts in Fish Guts and in Water Between the Three Experiments

**FIGURE 5:**
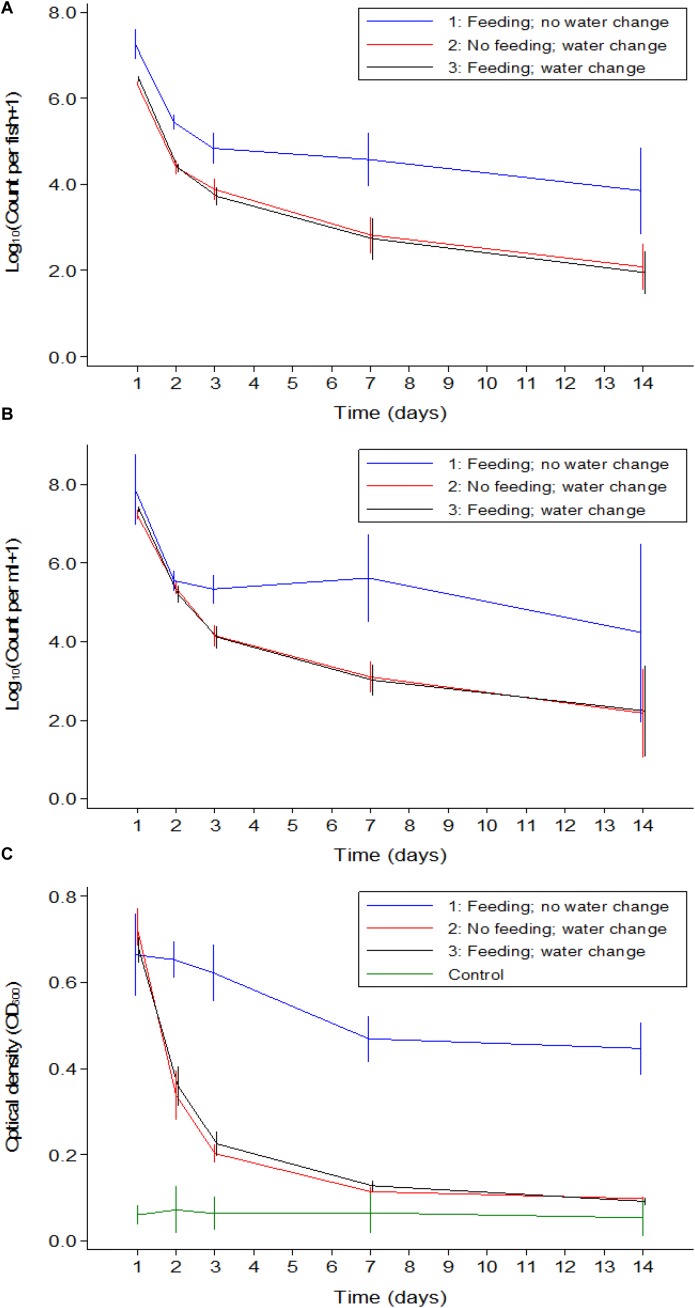
Comparison of *V. cholerae* counts (all strains combined) in tilapia guts **(A)**, in water **(B)**, and absorbance **(C)** between the three experiments overtime. Relevant statistical differences between experiments and time points are indicated in the text.

A comparison between the three experiments was made to distinguish if feeding or water renewal influenced the survival of *V. cholerae* in fish. In stagnant water (Experiment 1), *V. cholerae* counts dropped from 10^7^ to 10^5^ cfu/mL and from 10^7^ to 10^4^ cfu/intestine in fish. However, in Experiments 2 and 3 where water was changed, *V. cholerae* in water decreased from 10^7^ to 10^3^ cfu/mL and gut counts ranged between 10^6^ and 10^2^ cfu/intestine, with significant daily decreases (*p* < 0.05). When water was replaced daily with fresh sterile tap water in Experiments 2 and 3, *V. cholerae* counts were statistically similar both in water and intestines regardless of presence or absence of feeding (*p* > 0.05; Figure 5A,B). This indicates that in both water and tilapia intestines, feeding did not have any impact on concentrations of *V. cholerae* (*p* > 0.05, Figure 5). In contrast, higher *V. cholerae* counts were recovered in water and intestine when water was not changed, i.e., comparing Experiment 1 with Experiments 2 and 3 (*p* < 0.05; Figure 5A,B). This significant variation between the three experiments was due to water renewal.

Throughout the experimental period, *V. cholerae* counts in tilapia intestines were not statistically different between Experiments 2 and 3 (*p* > 0.05); however, these counts were significantly lower when compared with Experiment 1 at each time point (*p* < 0.01). Furthermore, in water, the comparison between experiments revealed that there was no significant difference between the three experiments during the two first days, but from day 3 to day 14, the change of water significantly influenced bacterial counts (*p* < 0.001).

The OD values of water was higher when the experiment was done in the same water as compared to when water was changed daily (*p* < 0.05, Figure 5C). Apart from day one post infection when OD values from the three experiments were statistically similar, the absorbance of water differed significantly in Experiment 1 when compared to Experiments 2 and 3 (*p* < 0.001), i.e., OD values in Experiments 2 and 3 were not statistically different (*p* > 0.05). We conclude that feeding had no significant influence on the OD values but the change of water did reduce the water turbidity overtime as the concentration of *V. cholerae* decreased.

### Transmission of *V. cholerae* Within Tilapia Populations

**FIGURE 6:**
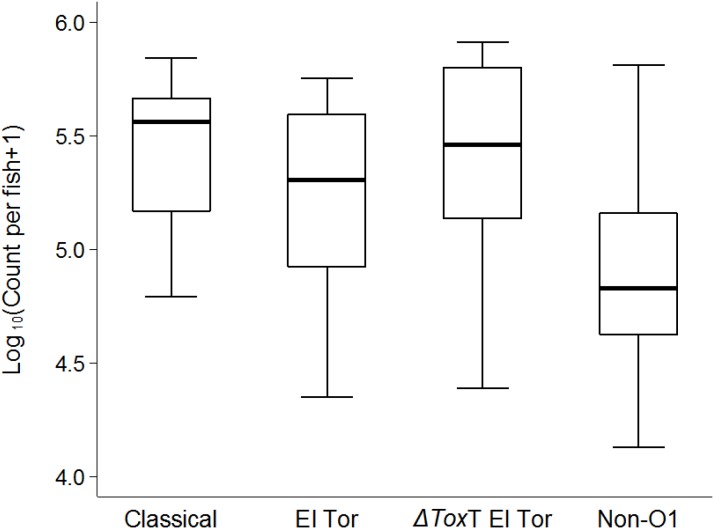
Transmission of *V. cholerae* from infected to naïve tilapia via excretions 24 h post cohabitation using box and whiskers plot. The thick line is the median and the box is the interquartile range and whiskers the range. Counts are from the two repetitions.

As we observed a stable survival of *V. cholerae* over 2 weeks in infected tilapia compared to the uninfected controls, a question emerged whether the bacteria could be transmitted from infected to naïve tilapia. After 24 h of cohabitation with infected tilapia (without feeding), average *V. cholerae* counts of 10^5^ cfu/intestine were observed in naïve fish that were initially tested free of *V. cholerae*. The concentration of *V. cholera*e found in naïve tilapia were similar for all four test strains (*p* > 0.05, Figure 6).

## Discussion

Experimental exposure model studies with *V. cholerae* in zebrafish suggest that *V. cholerae* can colonize fish guts and be transmitted among zebrafish populations (Runft et al., 2014; Mitchell et al., 2017). Our results in tilapia are consistent with the observations in zebrafish. High counts of the different strains of *V. cholerae* in tilapia intestines were observed 24 h after exposure as a sign of colonization. The sharp drop in *V. cholerae* counts in fish and water between 24 and 48 h post inoculum in all three colonization experiments could be due to natural shock of the *V. cholerae* bacterial cells attributable to changed environments, which may lead to a dormant state also known as viable but not culturable (Kamruzzaman et al., 2010). However, after day 2 to 3 post infection, concentrations of *V. cholerae* remained more or less stable, demonstrating adaptation and survival in the tilapia. Non-toxigenic strains, notably Δ*toxT* of *V. cholerae* O1 El Tor and the environmental non-O1 strain, were also able to colonize fish and persist over time. Intestinal colonization of *V. cholerae* in humans requires production of the cholera TCP, whose main virulence transcription activator is ToxT (Faruque et al., 1998; Sanchez and Holmgren, 2011). The fact that *tox*T mutants and non-O1 strains of *V. cholerae* were found in tilapia intestine over time is in accordance with observations in zebrafish (Runft et al., 2014) and suggests that TCP is not essential for *V. cholerae* colonization of fish. Moreover, studies have discovered a novel flagella-mediated cytotoxin MakA which is proposed to be involved in *V. cholerae* intestinal colonization in zebrafish (Dongre et al., 2018). *V. cholerae* strains used in our study are wild types and possess a flagellum, so the secretion of MakA protein associated with flagella could be involved in the colonization of tilapia.

In Experiment 1, where fish were kept in stagnant water and fed live brine shrimp (*A. salina*) free of *V. cholerae*, there were high *V. cholerae* counts in intestines and water together with high OD values. Since the infection dose (5 × 10^7^) was the same in all experiments, the higher concentrations of *V. cholerae* observed in Experiment 1 compared to findings in Experiments 2 and 3, in which water was changed daily, were thought to be associated with the continuous provision of brine shrimp, as their presence could enhance attachment and multiplication of *V. cholerae* in the fish intestine. The ADP-ribosylating cholix toxin in *V. cholerae* has been shown to play an important role in the survival of the organism in the aquatic environment and facilitates its attachment to crustaceans, notably the brine shrimp. Moreover, *V. cholerae* are known for their ability to attach to chitin exoskeletons of shrimp, copepods and other crustaceans that serve as substrate for their survival and multiplication (Huq et al., 1983; Tamplin et al., 1990; Hood and Winter, 1997; Patra and Mohamed, 2003). However, results in Experiment 3 where water was replaced on a daily basis and fish were fed with the same live feed rejected the hypothesis that brine shrimp could enhance colonization, because the change of water was found to be the only significant variable associated with *V. cholerae* concentrations.

Comparison of the three experiments shows that irrespective of feeding and water exchange, tilapia were colonized by environmental non-O1 *V. cholerae* as well as *V. cholerae* seventh pandemic El Tor (7PET) strains and were isolated beyond 2 weeks. This strongly suggests that in natural aquatic environments, where fish can live in stagnant or running water with presence of various feed items, tilapia may constitute a reservoir of toxigenic, and non-toxigenic strains of *V. cholerae.* It is worth noticing that the concentration of *V. cholerae*, i.e., 10^7^ cfu per ml water in the beakers, was higher as compared to concentrations that can be expected in natural aquatic environments during non-cholera outbreak periods (Senderovich et al., 2010). In contrast, there are little data available about the actual concentration of *V. cholerae* O1 in such environments. In a previous study, we did report that tilapia were able to live in raw sewage and were found to carry *V. cholerae* O1 (Hounmanou et al., 2016). Moreover, tilapia in our experiment were infected by immersion and we therefore used a similar dose of 10^7^ cfu per ml as in the zebrafish experiments (Runft et al., 2014; Mitchell et al., 2017) to ensure an uptake of the test strains. It is not known how many *V. cholerae* cells fish ingest in natural water systems. The high inoculum of *V. cholerae* used in this study could enhance colonization and may represent a limitation of our study. Nevertheless, even at low concentrations of *V. cholerae* similar to natural conditions, like from day 7 in all our experiments (10^3^ CFU/intestine), tilapia remained colonized, with the O1 El Tor biotype maintaining the highest numbers. Furthermore, the transmission experiment with naïve tilapia placed in beakers with tilapia carrying *V. cholerae* in the intestine showed that when the naïve tilapia were exposed to about 10^5.5^ cfu per mL water they became infected and had similar bacterial concentrations in their intestine after 24 h (Figure 6). It should be noted that we did not determine the *V. cholerae* concentration in the water in the transmission experiment (Figure 6) and that the stated expected concentration in the water of 10^5.5^ cfu per mL is based on values found at day 2 in Experiment 2 (Figure 3B), where infected tilapia were washed and transferred to beakers with fresh water. Overall, the results indicate that irrespective of the initial concentration of *V. cholerae* in water, tilapia can become colonized with *V. cholerae* and act as a reservoir for transmission and long-term survival.

Findings from Experiments 2 and 3 show that the concentration in water after 1 week was around 10^4^ to 10^3^ cfu/mL similar to what has been reported in the natural environmental waters (Senderovich et al., 2010). One week later, the concentration of *V. cholerae* was around 10^3^ to 10^2^ cfu/mL. Despite a low concentration of *V. cholerae* in water seen during the last 7 days and the continuous daily water renewal, the OD values of the water in the beakers remained higher than the OD values in the beakers of the control fish. This suggests that the increased OD water values were due to excreted material from the tilapia, i.e., stress-related discharges, probably due to the initial high concentration of *V. cholerae* in the water. *V. cholerae* are able to colonize tilapia over an extended time span, multiply in the intestine, and be excreted into the aquatic environment but a high initial concentration could be stressful for the fish. We therefore suggest further studies to explore lower infection doses administered possibly by gavage to ensure sufficient uptake. Moreover, even when the Classical biotype of *V. cholerae* was no longer detectable in tilapia intestines and in water, the optical density of water in those beakers remained higher than in the control beakers. This is consitent with observations in zebrafish that heat-killed *V. cholerae* still induced mild diarrhea in zebrafish (Mitchell et al., 2017). This further suggests that the discharges and water turbidity provoked by *V. cholerae* in tilapia is neither due to cholera toxin genes nor to viability or biotype of *V. cholerae* but probably caused by the stress generated by the high initial infection dose of *V. cholerae*. Furthermore, the flagella-mediated secretion of MakA cytotoxin was suggested as a source of toxicity and death in zebrafish infected with wild-type *V*. *cholerae* (Dongre et al., 2018).

The absence of Classical biotype *V. cholerae* after 1 week and the persistence of the seventh cholera pandemic biotype El Tor *V. cholerae* O1 strains is similar to findings in zebrafish (Runft et al., 2014) and consistent with the rare isolation or extinction of the Classical biotype in the ongoing cholera pandemic (Echenberg, 2011; Weill et al., 2017). The El Tor biotype and the non-O1 serogroup strains seem more fit in the fish gut (aquatic environment) than the Classical O1 biotype strains which may explain the increasing recovery of these strains in most contemporary environmental studies (Hounmanou et al., 2016; Bwire et al., 2018). The persistence of *V. cholerae* O1 biotype El Tor in tilapia and water is of public health relevance as it provides evidence of environmental survival of the current pandemic El Tor biotype strains where they can emerge from and cause epidemics. Furthermore, *in vitro* and *in vivo* experiments have demonstrated that in the presence of glucose, *V. cholerae* of the Classical biotype generates organic acids that inhibit their growth, while the growth of El Tor biotype is enhanced due to their ability to produce acetoin (2,3-butanediol), a neutral fermentation end product (Yoon and Mekalanos, 2006; Sengupta et al., 2017; Nag et al., 2018). It could therefore, be that carbohydrates present in the water of the beakers, e.g., droppings from the tilapia, did facilitate glucose metabolism of *V. cholerae*, resulting in loss of viability of the Classical biotype and survival of El Tor biotype. Such unfavorable conditions may also cause the strains, especially the Classical biotypes, to enter a dormant state, known as viable but not culturable (VBNC) (Bari et al., 2013; Xu et al., 2018), which may be one explanation as to why the Classical biotype strains were not detected after 1 week of the experiments.

The concentrations of *V. cholerae* in tilapia intestines correlated with those in the water. As the number of fish in the beakers decreased over time, *V. cholerae* counts in water decreased. This strong correlation (*p* < 0.0001) between counts in water and fish indicates that *V. cholerae* have reduced ability to multiply in clean water (Colwell et al., 2003) as compared to the intestine and is continuously excreted by the fish host. Thus, the main significant predictor of *V. cholerae* concentrations in water was the concentration of *V. cholerae* in the fish intestine. Moreover, the observed similar *V. cholerae* counts in water and in fish intestines in all experiments is likely attributable to the fact that the beakers used as aquarium provided a restrained space to the fish in a low volume of water (2 L). When *V. cholerae*-free tilapia cohabitated with infected tilapia in sterile water overnight, their intestine was also colonized, providing evidence of transmission. The transmission was not strain-dependent, as all the four test strains had similar transmission rates, demonstrating that toxigenic and nontoxigenic strains can equally be transmitted between tilapia populations and that *V. cholerae* can survive and amplify in tilapia, and also be disseminated from tilapia. Our findings are similar to reports in zebrafish models (Runft et al., 2014). Furthermore, the fact that naïve tilapia became infected in the transmission experiments substanciates again that tilapia were effectively and stably colonized by *V. cholerae* that then was disseminated between fish populations. Although only intestines were studied in our experiments, other organs like gills and skin could also play a significant role in the transmission process as they would provide nutrients favoring colonization of *V. cholerae*. However, previous studies have demonstrated that intestines are the main factors involved in colonization and transmission of *V. cholerae* in fish (Runft et al., 2014) and fish were washed twice with sterile saline by rubbing before the transmission experiment to remove external bacteria. The potential epidemiological importance of this study is that during a cholera outbreak, while all efforts are deployed toward containing the epidemics at human level, fish may serve as vehicle of dissemination of the bacteria in other areas, which may subsequently be hit by the same outbreak even when human patients are quarantined in the initial outbreak settings. The possible role of fish in transmitting *V. cholerae* is further supported by the findings that fish eating birds such as Great cormorants have been found to carry and disperse *V. cholerae* in space and time as they feed on infected fish and get colonized by *V. cholerae* (Laviad -Shitrit et al., 2017).

In summary, we have demonstrated that toxigenic *V. cholerae* O1 biotype El Tor and non-toxigenic strains of *V. cholerae* colonized the intestines of tilapia and were transmitted to naïve tilapia. This study provides answers to a hypothesis posed in a previous study (Halpern and Izhaki, 2017) that fish can be colonized by *Vibrio cholerae* and subsequently horizontally transfer *V. cholerae* to other fish within the same species, and probably to other fish species. This suggests that *V. cholerae* colonizes and persists in fish, and is transmitted between fish in aquatic environments, which may influence the epidemiology of cholera. Tilapia and other fish are potential reservoir hosts involved in the survival, excretion and transmission of *V. cholerae* in time and space. Cholera surveillance strategies may need to be updated accordingly including analysis of fish for the presence of *V. cholerae* O1 in aquatic environments. We furthermore suggest further studies to confirm the role of tilapia as an environmental reservoir host of *V. cholerae* O1 biotype El Tor using lower infection doses administered possibly by gavage to ensure sufficient uptake and limit stress to the fish.

## Data Availability

The raw data supporting the conclusions of this manuscript will be made available by the authors, without under reservation, to any qualified researcher.

## Ethics Statement

The present study was approved by the ethical review board through the ethical clearance certificate for conducting animal related research in Tanzania with ethical approval number SUA/CVMBS/018/07 (File submitted to editorial). Euthanasia of fish during the study and disposal of waste materials were performed according to instructions of the above-mentioned ethical approval.

## Author Contributions

YH designed the study, carried out the experiments in the laboratory, analyzed the results, and drafted the manuscript. RM supervised the experiments and critically reviewed and edited the original draft of the manuscript. TD participated in critical reviewing and editing of the manuscript. HM contributed to statistical analysis and data interpretation. JW provided the test strains and critical revision of the manuscript. JO and AD conceived the study and reviewed the manuscript. AD validated the data and supervised the study. All authors read and approved the final manuscript.

## Conflict of Interest Statement

The authors declare that the research was conducted in the absence of any commercial or financial relationships that could be construed as a potential conflict of interest.
